# Opinion: pelvic floor disorders: learning from chronicity & chronic care models

**DOI:** 10.3389/fgwh.2023.1006693

**Published:** 2023-06-19

**Authors:** Inès Osenge-Nyoyi Ongenda, Zelalem Mengistu, Douglas Tincello, Christopher Williams, Emma Pitchforth

**Affiliations:** ^1^ Primary Care Research Group, Department of Health and Community Sciences and Faculty of Health and Life Sciences, University of Exeter, Exeter, United Kingdom; ^2^ Obstetrics and Gynecology Department, University of Gondar, Gondar, Ethiopia; ^3^ Women's and Children's CBU, University Hospitals of Leicester NHS Trust, Leicester General Hospital, Leicester, United Kingdom; ^4^ Department of Health Sciences, College of Life Sciences, University of Leicester, Leicester, United Kingdom

**Keywords:** pelvic floor disorders, chronicity, chronic care models, incontinence, women-centered care, quality care

## Introduction

Pelvic floor disorders, used here as an umbrella term, encompass pelvic organ prolapse, urinary incontinence, fecal incontinence, and sexual dysfunction.

Pelvic floor disorders (PFDs) are a significant burden for women. In any country at least 10 per cent of women across all age groups are likely to live with urinary incontinence or pelvic organ prolapse (1). Until recently these disorders had been given limited consideration in low- and middle-income country contexts, but studies of prevalence are starting to increase (2). In Ethiopia, for example, estimates show that 12%–20% of women may be affected (3) and that living with these disorders can cause significant distress (4), adversely impact daily activities (5), social roles, economic wellbeing, and personal and sexual relationships (6). Pelvic floor disorders, associated partly with childbirth (7) and physical labour, are neglected globally, even in well-resourced health systems.

Pelvic floor disorders fall between major policy groupings and features in different specialities (gynaecology & obstetrics, care of the elderly, urology …) but has no place of its own. These disorders are sometimes treated through the lens of maternal and reproductive health (7), sometimes through general gynaecology care (8) and sometimes through urology and surgery (9). Additionally, globally, pelvic floor disorders such as urinary incontinence and organ prolapse are considered a disease of ageing (10). This does not capture fully the population affected, particularly in low- and middle-income countries where the population is primarily of reproductive age (3).

Evidence on treatments for incontinence and prolapse from high income settings show that conservative management including pelvic floor muscle training can be effective, with studies showing cure being reported up to six times more for urinary incontinence vs. non-intervention (11–13). Surgical options are appropriate for women who have exhausted conservative management but are associated with high rates of reoccurrence (14, 15). There is interest in whether similar models of conservative management would be appropriate and effective in lower-income country contexts. While welcoming an increase in attention to this neglected area and the potential for relatively low-cost interventions, we argue there is also an important opportunity to avoid seeking to merely replicate models from higher income countries.

Typically conceptualised as an acute problem, we consider whether there is useful learning from the concept of “chronicity” and understanding of chronic diseases and chronic care management to inform how pelvic floor disorders may be better framed within health systems, policies, and service delivery. In line with the integration of people-centred health services (16) we begin by considering what is known about women's experiences of pelvic floor disorders and preferences for care before considering the value or otherwise of conceptualising as a chronic condition and potential implications for models of care. To reflect the limitations of the current model, our argument is accompanied by a case study from Gondar, Ethiopia, where one of the authors (ZM) is based.

## Pelvic floor disorders in gondar university hospital (Ethiopia): reflections on current limitations of models of care

### Management of pelvic floor disorders in gondar university hospital

In Gondar University Hospital, despite limited provision of non-surgical treatments, an array of advanced surgical procedures is available as the hospital is one of the key training sites for urogynaecology and pelvic reconstructive surgery subspecialists. Staff manage 400 patients with pelvic floor disorders (PFDs) per year and have approximately 300 patients waiting for treatment.

### The main challenges in the model currently at the University of Gondar and in Ethiopia more broadly

Pelvic floor disorders (PFDs) have no specific assigned category within the health system. However, people in the Maternal and Child health Directorate in the Federal Ministry of Health are responsible for it at national level. Unfortunately, funders consider PFDs as a condition treated only once. Therefore, donors only support surgery leaving not funds for continued care. It has created problems for both the providers—for further management and follow-up, as well as for the women as they seek to access further care.

This “once and done” thinking gives false reassurance and perpetuates substandard care.

### Arguments about seeing pelvic floor disorders as a chronic disease in Ethiopian context

Women with pelvic disorders in Ethiopia are relatively young with a predisposition for risk factors from an early age. They are at a risk of malnutrition for example, which affects the pelvic bone development and later causes them to have difficult labour, possibly at home by a traditional birth attendant. Early marriage and multiple deliveries, short interval deliveries, and engagement in heavy duty activities in their daily lives soon after delivery also put them at risk. Women suffer in silence for an extended amount of time or for life, as they do not understand what has happened to their body and are ashamed to share their difficulties with their partners or anyone else for fear of stigmatization and discrimination (17). Furthermore, even if they want to visit health facilities, they are not empowered to do so.

Recently, the Ministry of Health and funders have recognized the burden of PFDs and have started mobilizing women with PFDs, mainly pelvic organ prolapse. Women are encouraged to receive treatment at their nearby Hospital where gynaecologists perform vaginal hysterectomy indiscriminately. However, this is making women with PFDs suffer for life and feel helpless and hopeless as it is an extensive procedure with a considerable impact in their life. In addition, since this is taken as a one-off treatment, they don't get the follow-up attention and assistance that they require after their first procedure.

Thus, taking out PFDs from maternal health and disease of old age, and categorizing under chronic disease or long-term conditions might allow those women to get the attention they deserve from the health system as well as from global funders and donors.

## Women's experience of pelvic floor disorders - how does this compare to how it is placed in policy and systems?

### Low vs. high-income countries

When it comes to healthcare providers involved in care there is a clear difference between low- and high-income countries. In high-income countries, historically people were looking at disease through an organ and system approach thus focusing on the reproductive and/or genital system. This dates back to the 18th century through Descartes. The patient is usually under the care of the primary care doctor and/or if the condition is advanced, a gynaecologist or a uro-gynecologist. This approach creates problems in low- and middle-income countries as health systems do not have the workforce to be speciality driven and often rely on external donor funding and programmes. These in turn often have a disease-specific focus which leave pelvic floor disorders neglected.

According to Bernell & Howard (18) there is not a single, accepted definition of “chronic disease” which implies that health professionals and policymakers do not necessarily agree on which diseases should be included (19).

In the context of this paper, we define pelvic floor disorders as chronic conditions given what we know about urinary incontinence and organ prolapse presentation: gradual disease, limitations on daily activities, and need for ongoing medical attention or self-management. Thinking about pelvic floor disorders differently may be useful to understand women's lived experiences and to inform health service and system design.

### Medical community

In the medical community, we can argue that chronic disease confers a specific advantage. In high-income countries, it means closer monitoring and various health promotions initiatives (from social prescribing to increased screening and more regular appointments). The downside is that those initiatives can sometimes lead to over medicalisation, particularly when it comes to surgical interventions (20). There is a wealth of literature with regards to the model of care for those suffering from chronic conditions (21) which are not necessarily available nor as comprehensive for those suffering from pelvic floor disorders particularly urinary incontinence and organ prolapse. In high income countries there is also evidence that the chronic care model can be a useful and sustainable tool to use to improve outcomes (22).

### Social sciences

In social sciences (23), the literature about chronic illnesses is centred around the need for better integration of current analysis around chronicity and the necessity to feature more predominantly the voice and experience of the chronically ill but also to dissect the current understanding of chronic disease—or should we say the various definitions of concepts around chronicity from chronic illness to chronic disease or chronic care. Furthermore, there is also a debate to better integrate the social context, impact and personal views of the patient when thinking about care. Social scientists are attempting to add those elements to current disease-based thinking and those arguments are particularly relevant in our scenario given the broadness and differences of the conditions under the umbrella term, “pelvic floor disorders”, and the varying impact they can have on women. Integrating evidence from social sciences can only enhance and improve understanding of the journeys of women affected.

## If we accept that pelvic floor disorders do share some of the features, what might we learn from chronic care models?

### Gender

Historically, there was little place for intersectionality or even more generally, a desire to take into account gender when developing research (24). Health systems and care model design have typically incorporated neither gender nor sex despite evidence highlighting that both impact health seeking experience, care provision and outcomes (25, 26). In a somewhat vicious cycle, the lack of attention to gender in health systems and services designs contributes to a recognised neglect for women's health, beyond reproductive health (27).

The fact that women's experience is not taken into account, especially for the design of systems where they represent 50% of the beneficiaries and care pathway where they can represent more than 80% of those affected, is a clear consequence of how health inequities can persist and flourish (28).

As we call for the creation of a new way to care for patients suffering from pelvic floor disorders—through a pelvic floor disorders chronic or long-term care model, we suggest that this should involve considerable input from women.

### High-quality woman-centred coordinated care

Woman-centred care refers to a philosophy which emphasises woman's individual needs when it comes to their healthcare needs while women-centred care is a philosophy which is used to guide service provision by putting women at the centre of care. Women-centred care and woman-centred care lead to better understanding and outcomes for women needing maternity services (29) but have been given limited attention beyond maternity and reproductive health care (30, 31). We argue that care models for pelvic floor disorders must put women at the centre and be responsive to their specific needs, preferences, and behaviours. We welcome additional research relating to women-centred care in gynaecology and female urology.

Coordinated care refers to “the deliberate organization of patient care activities between two or more participants involved in a patient's care to facilitate the appropriate delivery of health care services” (32). Several studies about the care women affected by pelvic floor disorders receive have noted how fragmented it was (33–35). In higher-income countries, there has been a clear need and call for care that is better integrated (35). Coordinated care is implemented for conditions such as diabetes or hypertension, but there is still work to do (36) particularly between integrating primary and specialist care (37). In lower and middle-income countries, there is also a clear need for better coordination of care and continuity of care. In countries where specialist healthcare needs—particularly outside of childbirth care, are met by the funding of external donors and programmes, fragmentation of care is the norm rather than the exception (38). Additionally, health systems financing decisions have enhanced this fragmented care pathway (particularly user fees, community health-based insurance and increasing cost of out of pocket expenditures) (39) thus further challenging an already complex issue (40). Moreover, it is important to advocate not only for access to coordinated care but also access to excellent quality of care. The two should not be dissociated for women suffering from pelvic floor disorders (41).

Care coordination is an essential component for women suffering from pelvic floor disorders, given the variety of people (patient, family, primary care doctor, pharmacists, sometimes surgeons, physiotherapists, nurses) involved in the management of those conditions. Whichever pelvic floor disorders chronic care model is adopted, it should ensure that it is woman-centred and provides excellent coordination care thus reducing fragmentation of care and improving experience and outcomes.

### Specialist/generalist and role of primary care

Studies have shown that there is still a disconnect between specialist and generalist care provision thus furthering a fragmented approach to care (42). Unfortunately, there has been little literature on how to better integrate specialists and generalists in lower and middle-income countries and, even fewer examples of studies depicting successful integration as opposed to merely advocating for more integration (37).

**Figure 1:**
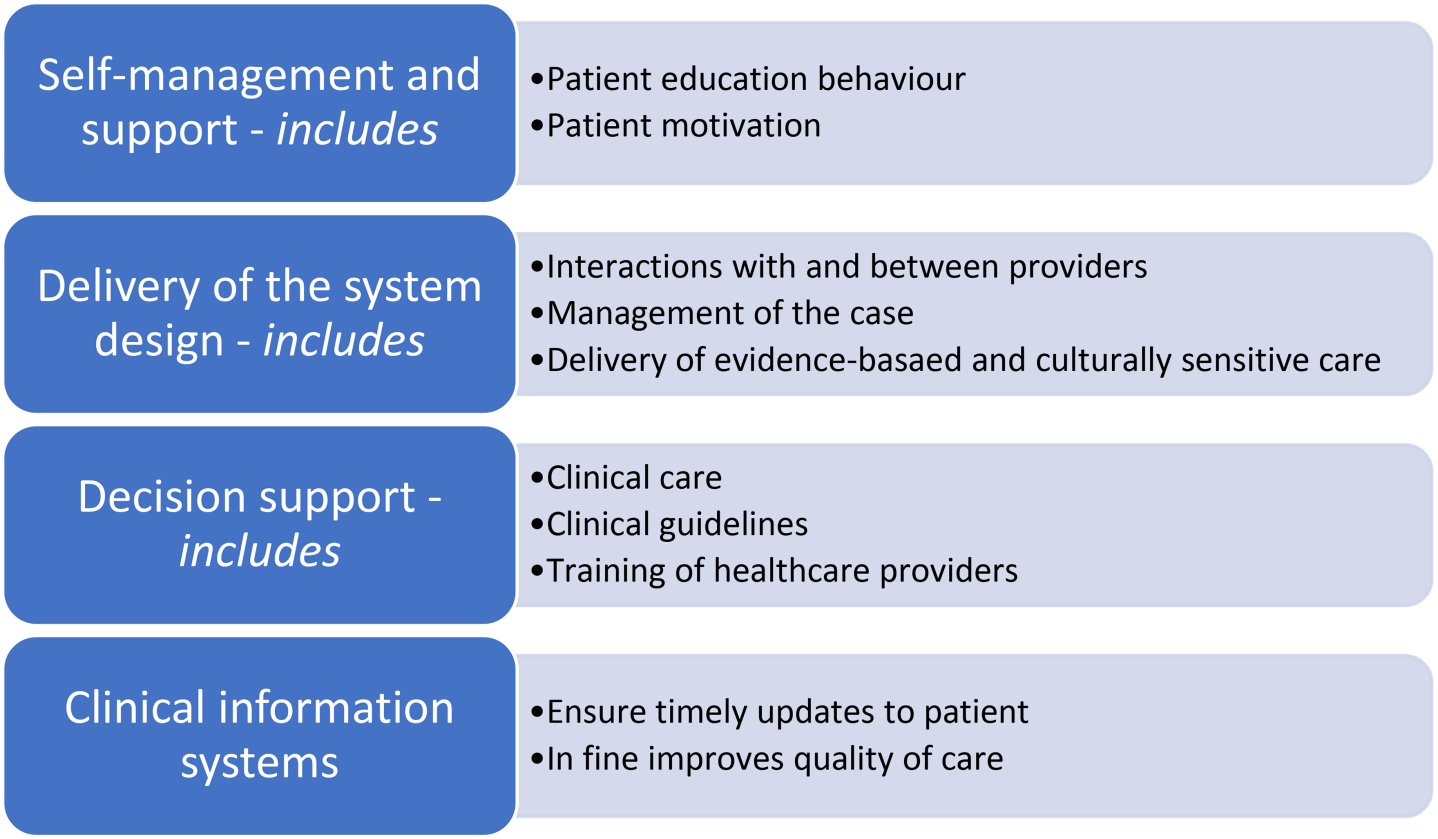
Example of health system design for chronic conditions [via (43)].

As seen in Figure 1, if we look at a current health system design for chronic care illnesses such as diabetes, hypertension or asthma, despite the differences when it comes to the pathologies and clinical management, there are still several similar characteristics (43). They are led by the community which provides resources and policies and have at the helm the primary care provider as the focal point for the team of healthcare providers.

At the system level, it usually encompasses elements shown in Figure 1. Data have shown that even if not all components of the chronic care model are available and utilised, there is still an increase in the quality of care that patients are receiving and a lesser chance to need acute care (44).

If we look at existing care models, currently, pelvic floor disorders do not fully sit within an acute care model nor a chronic care model to the detriment of those who are affected. Although severe case of urinary incontinence and prolapse in high-income countries and low-income countries (if care is available), are managed using an acute care model—with surgical interventions and inpatient stay -, the milder cases are managed in high-income countries such as the UK at the community level with a primary care physician approach (33). However, that approach does not currently fit the chronic care model as we understand it, due to a lack of comprehensive and coherent strategy. This is due to lack of funding, paucity in education, lack of public awareness and difficulty for various healthcare providers to come together and collaborate regularly for the betterment of their patients.

This strengthens our argument that pelvic floor disorders should be integrated within a chronic care model, which would be primary care-led but women-centred thus allowing for significant improvement for the lives of women affected.

This chronic care model could be established, with appropriate adaptations, for women suffering from pelvic floor disorders in high and low and middle-income countries (see case study from Ethiopia) where a primary care rather than acute care model may align better with workforce composition. Now is the time to consider pelvic floor disorders more holistically.
